# Potency of Combining *Eucalyptus camaldulensis* subsp. *camaldulensis* with Low-Dose Cisplatin in A549 Human Lung Adenocarcinomas and MCF-7 Breast Adenocarcinoma

**DOI:** 10.3390/medicines7080040

**Published:** 2020-07-22

**Authors:** Mohamad Nasser, Raghida Damaj, Othmane Merah, Akram Hijazi, Christine Trabolsi, Nour Wehbe, Malak Nasser, Batoul Al-Khatib, Ziad Damaj

**Affiliations:** 1 Plateforme de Recherche et d’Analyse en Sciences de l’Environnement (EDST-PRASE), Beirut P.O. Box 5, Lebanon; mohamed.nasser@ul.edu.lb (M.N.); rdamaj82@hotmail.com (R.D.); nourwehbi6@gmail.com (N.W.); malak-nasser1994@hotmail.com (M.N.); batool.khatib93@hotmail.com (B.A.-K.); damajz@hotmail.fr (Z.D.); 2 Laboratoire de Chimie Agroindustrielle, Université de Toulouse, INRA, 31030 Toulouse, France; 3 Département Génie Biologique, IUT A, Université Paul Sabatier, 24 rue d’Embaquès, 32000 Auch, France; 4 Faculty of Medicine, Lebanese University, Beirut P.O. Box 5, Lebanon; christinetrabolsi9@gmail.com

**Keywords:** eucalyptus, cisplatin, antitumor effect, cell viability, antioxidant activity, phenol content

## Abstract

**Background:** Lung and breast cancers are common in the world and represent major public health problems. Systemic chemotherapy is an effective way to prolong survival but it is associated with side effects. Plants are used as traditional treatments for many types of cancers, mostly in combination with chemotherapy. We investigated the antitumor effect of ethanolic (EE) and aqueous (AE) extracts of *Eucalyptus camaldulensis* on human alveolar adenocarcinoma basal epithelial cells (A549) and breast adenocarcinoma cell line (MCF-7) and checked the synergistic effect of the combination with low-dose cisplatin (CDDP). **Methods:** AE and EE were characterized for their secondary metabolites including content of phenol and antioxidant activity of both extracts. Cell viability was tested by the neutral red assay and MTT. Combinations of extract with low-dose CDDP on A549, MCF-7 cells, and normal cells peripheral blood mononuclear cells was used to study cell viability. **Results:** AE contains higher level of active constituents than EE. Higher antioxidant activity was observed in AE. Both extracts showed cytotoxic activity on A549 and MCF-7 cells. Moreover, combining *E. camaldulensis* with low-dose CDDP increases significantly the cell death of treated cells in comparison to those treated with CDDP alone. **Conclusions:** Our results highlight a new therapeutic concept that combines *Eucalyptus camaldulensis* with low-dose CDDP to treat lung and breast adenocarcinoma.

## 1. Introduction

Cancer is a disease caused by the transformation of cells that become abnormal and proliferate excessively. These disordered cells sometimes end up forming a mass called a malignant tumor which promotes tumor formation and metastasis.

Cancer is a major public health problem worldwide and it is the second leading cause of death in the United States. In 2018, about 1,735,350 new cancer cases and 609,640 cancer deaths are projected to occur in the United States [1].

The most common causes of cancer death are cancers of the lung, breast, and colorectal in females whereas cancers of lung, colorectal, and prostate in males. These cancers account for about half of all cancer deaths, with more than one-quarter due to lung cancer [2].

For a few decades, lung cancer has been and remains the foremost common danger within the world with an assessed 1.6 million new cases per year (12.7%). It is additionally the driving cause of cancer-related mortality with an evaluated 1.37 million deaths per year [3].

Routine treatments such as chemopreventive agents are characterized as compounds anticipating the event of harm by restraining the enactment of a carcinogen, repressing its interaction with cellular macromolecules, or by deactivation and clearance of the carcinogen, or to control or switch the damage caused by a carcinogen. These chemopreventive agents which will decrease the frequency and mortality of cancer may be displayed in plants, vegetables, and natural products [4].

There are several treatments for lung and breast cancer, including surgery, radiotherapy, and chemotherapy. According to the circumstances, each of these treatments may be undertaken with either a ‘radical’ or ‘palliative’ intent [5,6].

About 70% of lung cancer patients have locally progressed or metastatic disease at diagnosis, and chemotherapy may be there as it was the treatment of choice. Chemotherapy may help decrease or slow the growth of locally advanced or metastatic NSCLC. It can also help in controlling the symptoms of some patients [6]. Thus, the importance of chemotherapy in the treatment of NSCLC based on the use of chemotherapy after surgery: For an early stage of NSCLC (non-small-cell lung carcinoma), chemotherapy after surgery may help reduce the risk of recurrence of cancer. Chemotherapy is usually started within eight weeks after surgery. Chemotherapeutic combinations tend to work better than simple medications. Physicians usually combine CDDP or carboplatin with at least one other medicine such as paclitaxel (Taxol) and docetaxel (Taxotere) [6].

Chemotherapy with radiotherapy: Chemotherapy is sometimes given before or after radiotherapy. It can help get rid of NSCLC at an early stage in people who cannot have surgery. These treatments can also prolong the survival of some people with a small NSCLC, even if they are not likely to be cured of their cancer [7].

However, the efficacy of chemotherapy in patients with advanced lung cancer is extremely limited, due to drug resistance and toxic side effects of drugs, which results from the genetic and epigenetic regulation of various critical genes [8].

Breast cancer remains the foremost common threatening tumor in ladies, in spite of advancements in determination and treatment. Even when treated early, about 50% of patients with breast cancer still encounter backslide and metastasis. For these patients, an efficient and comprehensive treatment is required, counting systemic chemotherapy, endocrine treatment, focused on targeted treatment. Systemic chemotherapy is one of the foremost viable treatment choices for repetitive metastatic breast cancer (rMBC), in spite of the fact that there is no gold standard regimen. Numerous chemotherapy regimens that incorporate anthracycline combined with Taxus are confined by total cardiac poisonous quality and sedate resistance. Docetaxel has been prescribed as a first-line medication for rMBC [9].

Treatment of lung, breast, and colon cancer cells with PTC-209 (potential compound a small-molecule Bmi-1 inhibitor) (1 and 2.5 μM) for 48 h showed no caspase-3 activation, but a decrease in the cell number below the seeding level suggests that PTC-209 reduces cellular viability probably through inhibition of cell proliferation and induction of cell death via a caspase-3-independent mechanism. The molecular mechanism analysis revealed that PTC-209 significantly inhibited the signal transducer and activator of transcription 3 (STAT3) phosphorylation by decreasing the expression level of gp130 (glycoprotein 130) as early as 30 min post-treatment [10].

The adequacy and security of the docetaxel and lobaplatin (DL) and docetaxel and gemcitabine (DG) regimens for treatment of repetitive and metastatic cancer were watched for the primary time. Based on the results, both of these combined chemotherapy regimens are viable, treatment-related side impacts are passable, and either can be utilized for viable treatment for progressed cancer [11].

However, these treatments induce undesirable effects and are thus little tolerated by most patients. Scientific research aims to find effective treatments with a better safety profile.

Medicinal plants have diverse and beneficial activities for several types of diseases such as cancer, bacterial infections, diabetes, and inflammatory diseases. Medicinal plants are the source of a wide variety of natural products such as phenolic acids and flavonoids which are very interesting for their antioxidant properties and antitumor effects. Therefore, naturally-derived compounds are considered to have less side effects compared to current medications such as chemotherapy [12]. Numerous plant species are as of now being utilized to treat or anticipate the advancement of cancer. Several analysts have recognized species of plants that have illustrated anticancer properties [13]. Eucalyptus is one of those medicinal plants widely distributed in Australia. Eucalyptus species are commonly encountered in the Mediterranean region. These species are traditionally used in medicine as analgesic, anti-inflammatory, and antipyretic remedies for the symptoms of respiratory infections, such as cold, flu, sinus congestion for diarrhea, and as an astringent in dentistry [14]. Moreover, the Eucalyptol of Eucalyptus species has been used in the pharmaceutical, cosmetics, food, and medicinal purposes [15].

Eucalyptus species have been utilized for the treatment of illness. In expansion, the gum that is gotten from the tree by making cuts within the trunk of *E. citriodora* is utilized for the treatment of diarrhea and bladder inflammation, and is connected to cuts and scraped spots and has chemopreventive impacts [16].

The Eucalyptus is a widely grown tree, well known for the production of essential oils, with high biological activities used in various fields. Its leaves also provide extracts used in cosmetic formulations [17].

The essential oils have been used for pharmaceutical and medicinal purposes, and several studies have reported that these oils displayed multiple pharmacological activities, including antibacterial, anti-inflammatory activities, antitermitic activity, larvicidal and mosquito repellent activities, as well as antioxidative and antiradical activities [18].

In Australia, Eucalyptus leaves were traditionally used to heal wounds and fungal infections. They are also used in the treatment of respiratory diseases, such as the common cold, influenza, and sinus congestion. In Africa, the powder of barks has been indicated as insecticide. In addition to their uses in folk medicine, many studies demonstrated analgesic, expectorant, anti-inflammatory, and antimicrobial properties from the leaves of Eucalyptus [19].

Eucalyptus species are also important in the forest area of Tunisia where the most abundant species are *Eucalyptus camaldulensis* and *E. cineria* [17].

In addition, the antiproliferative effect of *E. citriodora* resin (ECR) on human hepatoma HepG2 cells was evaluated; so that the results from the MTT assay showed that water extracts of ECR (WEECR) in the dose range of 0–500 µg/mL displayed stronger cytotoxic effects on HepG2 cells than other organic solvent extracts of ECR. Moreover, the WEECR treatment has an apoptotic response in HepG2 cells, with an increased Bax/Bcl-2 ratio and activation of caspase-3 [19]. Cisplatin (CDDP) is a well-known chemotherapeutic drug which has been used for the treatment of numerous human cancers including bladder, head and neck, lung, ovarian, and testicular cancers [20].

The combination of a Chinese herbal medicine and CDDP may represent a novel approach in the treatment of NSCLC and thus offer a new target for chemotherapy [4].

The *E. camaldulensis* flower essential oil decreases melanin synthesis, inactivates protein kinase A (PKA) and mitogen-activated protein kinase (MAPK) signaling pathways, and inhibits tyrosinase activity which has an antioxidant activity, as well as decreases melanogenesis in melanoma cells by [18].

The essential oils of *E. camaldulensis* may be potentially suitable for controlling mosquitoes at the larval stage. The volatile fractions isolated from *E. camaldulensis* leaves collected in Kurdistan contained critical and broadly used flavor and scent fixing. Most constituents, basically 1,8-cineole and other oxygenated monoterpenoids, are naturally dynamic, showing well-known antibacterial, bronchodilatory, anti-inflammatory, and pain-relieving impacts. They illustrated that the inward breath of vapors from a hot watery implantation of *E. camaldulensis* taking off in diseases, is maintained by logical prove [21].

The antimicrobial potential of the basic oils extracted from seven Eucalyptus species was higher against the Gram-positive microscopic organism than the Gram-negative ones and two sorts of parasites. These fundamental oils were exceptionally successful and may be used in solutions, makeup, nourishment, and flavors businesses. The combination of a Chinese herbal medicine and CDDP may represent a novel approach in the treatment for NSCLC and thus offer a new target for chemotherapy [4].

In this study, our aim is to produce a chemotherapeutic agent that will induce a synergetic effect by decreasing the dose of CDDP and combine it by natural constituents of Eucalyptus. Therefore, we investigated the effect of a combination of low doses of CDDP (cis-dichloro-diamine-platinum) with two different extracts (ethanolic and aqueous) of Lebanese Eucalyptus species on A549 and MCF-7 (Michigan Cancer Foundation-7) cell lines, and on normal cells (PBMC).

## 2. Materials and Methods 

### 2.1. Plant Material

Leaves of Lebanese *E. camaldulensis* subsp. *camaldulensis* were collected at flowering from Beirut (altitude: 0 m) in February 2017. The fresh leaves have been washed with distilled water to eliminate all impurities, and then they were kept in the oven at 50 °C for two days in order to dry. The dried leaves were grinded manually using mortar and pestle to be transformed into powder. The powder was preserved at 4 °C in plastic containers away from humidity, heat, and light for further uses.

### 2.2. Preparation of Crude Extracts

Extraction was done using the ultrasound machine in order to prepare the crude extracts. Therefore, 20 g of the dried powder were weighed and 100 mL of the selected solvent (water or ethanol) were added to them. The solution was then put in a 500 mL Erlenmeyer flask which was then put in a sonicator (ultrasound generating apparatus, Hielscher Ultrasound Technolgy, Teltow, Germany) at 60 °C for 30 min. Then, the extracts were subjected to centrifugation for 5 min at 1200 rpm which is followed by the Buchner funnel under reduced pressure to remove insoluble residues. After that, for the aqueous extract; it was put at -80 °C then subjected directly to the lyophilizer (Fischer, Illkrich, France) for three days to remove water and transform them to powder. While the ethanolic extract was put at -80 °C, subjected first to the rota-vapor method to remove ethanol and then water was added to remove the extract that is stuck to the balloon, then to the lyophilizer to remove the water and get the dry powder. Finally, the dried powder was preserved in plastic falcons in a desiccator for further use.

The obtained residue was weighed and the percentage yield was determined according to the following formula:

$$Yield\;\left(\%\right)=\frac{\mathrm{Obtained}\;\mathrm{mass}\;\mathrm{of}\;\mathrm{sample}\;\mathrm{extract}}{\mathrm{Initial}\;\mathrm{mass}\;\mathrm{of}\;\mathrm{powder}\;\mathrm{sample}}*100$$

### 2.3. Chemical Analysis

The qualitative tests (phytochemical screening).

In order to study the chemical composition of prepared plant extracts, a qualitative detection of secondary metabolites has been applied (Table 1).

### 2.4. Chemical Quantifications of Secondary Products

#### 2.4.1. Dry Matter Test

One gram of powder was put in a beaker and then placed in the oven at 100 °C for 24 h. The percentage of dry matter was calculated according to the following formula:

$$\%\mathrm{MF}=\%\mathrm{Dry}\;\mathrm{Matter}=\frac{\left(M2-M0\right)}{\left(M1-M0\right)}*100$$

where M0 is the mass of beaker alone, M1 is the mass of beaker + powder (before being placed in the oven), and M2 is the mass of beaker + powder (after being placed in the oven).

#### 2.4.2. Soluble Sugar Test 

One hundred milligrams of powder (before extraction) were weighed. Marication was done in 5.25 mL of 80% ethanol for 12 h. Then, centrifugation was done at 4000 rpm for 10 min. The supernatant contains the sugar, so it was taken and diluted 50 times to obtain solution A. After that, solution B was prepared and contains 1 g of anthrone that precipitates the sugar and 500 mL of concentrated sulphuric acid. Two milliliters of solution A and 4 mL of solution B were placed in a tube, then subjected to vortex for 1 min, then put at 92° C for 8 min. Then, the tube was put in the dark for 30 min. Finally, the absorbance was measured by spectrophotometry at a wavelength of 585 nm.

#### 2.4.3. Total Phenolic Content (TPC)

The estimation of the total phenolic content was done using the Folin-Ciocalteu reagent method.

Ten milligrams of the extracted powder and 10 mL of distilled water was put in a tube for the aqueous extract, while for the ethanolic extract, 10 mL of ethanol was added. Then, the solution was subjected to vortex for 1 min. Then, 40 µl of the prepared solution was taken, and 3.16 mL of distilled water + 200 µL of Folin reagent were added to it. Vortex was done to obtain solution A. After that, 20% of Na_2_CO_3_ was prepared. In addition, 600 µL from it were taken and added to solution A. Vortex was done again for 1 min. The prepared solution was put in a water bath at 40° C for 30 min. Finally, after it has been removed from the water bath and cooled down, the absorbance was measured at a wavelength of 765 nm.

The results were expressed in milligrams of gallic acid equivalent (GAE) per gram of dry weight of plant powders:

TPC = GAE × V ×D/M

where GAE is the gallic acid equivalence (mg/mL), V is the volume of the extract (mL), D is the dilution factor, and M is the mass (g) of the pure extract of the plant.

### 2.5. DPPH Scavenging Assay for Estimation of Antioxidant Activity

1,1-diphenyl-2-picrylhydrazyl (DPPH) is characterized by a stable free radical by virtue of the delocalization of the spare electron over the molecule as a whole, so that the molecule does not dimerize, as would be the case with most other free radicals. The delocalization of electron also gives rise to the deep violet color, characterized by an absorption band in the ethanol solution centered at about 517 nm. When a solution of DPPH is mixed with that of a substrate that can donate a hydrogen atom, then this gives rise to the reduced form with the loss of this violet color and the appearance of yellow color instead [22].

### 2.6. Evaluation of Antioxidant Activity

For each of the two extracts (aqueous and ethanolic), the antioxidant activity was determined by using the free radical 2.2-diphenyl-1-picrylhydrazyl (DPPH) scavenging activity. To do so, 0.0012 g of DPPH was dissolved in 50 mL of methanol; so that the concentration of DPPH is 6 × 10^−3^ M.

For the aqueous extract; 5 mg of powder was dissolved in 1 mL of methanol, while for the ethanolic extract; 5 mg of powder was dissolved in 0.5 mL of methanol + 0.5 mL of distilled water. After that, 50 µL of this prepared solution were taken and put in a tube, then 2 mL of the prepared DPPH solution were added to them. Then, the tubes were put in the dark for 30 min. Finally, the absorbance was measured at 515 nm. The antioxidant activity was calculated according to the following equation:

% Antioxidant activity =100×(ABS control− ABS sample)/ABS control

The ABS control is the absorbance of DPPH + solvent and ABS sample is the absorbance of the DPPH + sample. Where the control was prepared by taking 2 mL of the prepared DPPH solution (DPPH + methanol) without the extract.

### 2.7. Culture and Treatment of Cancer Cell Line

#### 2.7.1. A549 Cell Line

**Figure 1:**
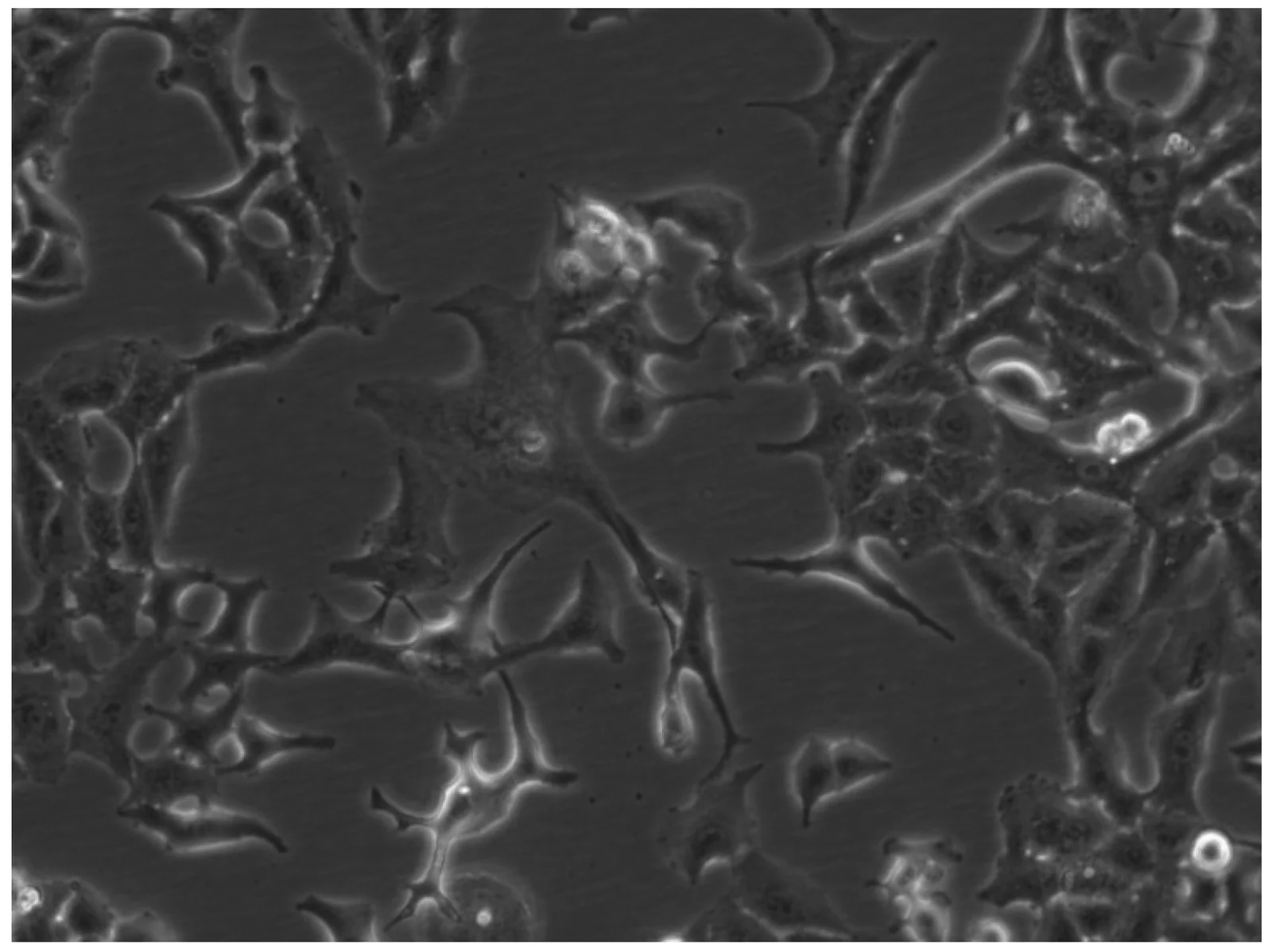
A549 cells captured at the American University of Beirut (AUB Lab).

Human NSCLC cell lines, epithelial lung carcinoma, and A549 obtained from ATCC, Manassas, VA, USA, were grown in non-coated T75 culture flasks as shown in Figure 1. Cells were filled with a complete medium: Dulbecco’s modified eagle medium (DMEM), supplemented with 10% fetal bovine serum (FBS), 1% penicillin/streptomycin (PS) in a humidified 5% CO_2_ atmosphere at 37 °C. The medium was changed every third day and the cells were harvested and separated using 0.05% trypsin when they have grown to subconfluence. They were transferred to a 96-well plate (5 × 10^4^) cells/well to study the cytotoxicity of *E. camaldulensis* and CDDP on A549 cells. 

#### 2.7.2. MCF-7 Cell Line

MCF-7 cell line obtained from ATCC, Manassas, VA, USA, is a widely studied epithelial cancer cell line derived from breast adenocarcinoma, has characteristics of differentiated mammary epithelium. These cells grow adherently as a monolayer, and were cultured in the same manner as A549.

#### 2.7.3. Peripheral Blood Mononuclear Cells (PBMC)

Human peripheral blood mononuclear cells (PBMCs) obtained from ATCC, Manassas, VA, USA, comprising lymphocytes (B-cells, T-cells, and NK-cells), monocytes, and dendritic cells, were frequently used for the evaluation of immune responses.

#### 2.7.4. Preparation of the Tested Concentrations

For the ethanolic extract, the powder was dissolved using vortex in the DMSO solvent, such that the DMSO concentration does not exceed 0.5% or else it will be toxic to the cells, while for the aqueous extract, the powder was dissolved using vortex in Dulbecco’s modified eagle medium (DMEM). The two extracts were filtered by a filter of diameter of 0.2 μm. The prepared concentrations for both ethanolic and aqueous extracts were: 75, 150, 300, 600, and 900 μg/mL.

For CDDP, the tested concentrations were prepared from a stock solution (1000 μg/mL), the obtained treatment test concentrations were: 0.5, 1, 2, 4, 8, and 12 μg/mL.

Combinations of *E. camaldulensis* extracts and CDDP were also prepared for each extract.

#### 2.7.5. Treatment of the Cells

A total of 70% to 80% confluent cells were seeded in a 24-well tissue culture microplate at the density of 100 × 103 cells/well and then treated with the prepared concentrations of *E. camaldulensis* and CDDP for 24 h. Evaluation of the anticancer activity was performed by measuring the cell viability of the A549, MCF-7, and PBMC by the neutral red assay after transferring the cells into a 96-well plate. Or, cells were directly seeded in a 96-well plate at a density of 10 × 10^3^ cells/well and then treated with the prepared concentrations of *E. camaldulensis* and CDDP for 24 h; where cell viability of the A549 was assessed by the MTT Assay.

#### 2.7.6. Isolation of Peripheral Blood Mononuclear Cells (PBMC), Ficoll Pague Isolation

Fifteen milliliters of blood were collected from a donor, and then 15 mL of PBS were added to them with a proportion (1:1). Fifteen milliliters of ficoll were added to the 30 mL of the prepared diluted blood, with a proportion (1:2). Centrifugation was done for 30 min at 400 RCF, where T (temperature) was 20 °C. The PBMC layer was removed and taken as a buffy white coat. PBMC was washed by 10 mL PBS. Centrifugation was done again for 15 min at 200 RCF to remove PBS. Another wash was done by 15 mL PBS to further purify the cells. Centrifugation was done again for 15 min at 200 RCF to remove PBS. The pellet containing the PBMC was taken, where the cells were resuspended in RPMI. PBMC was counted and finally seeded in wells. Finally, PBMC was subjected to treatment with *E. camaldulensis* and CDDP in the same way as A549, where the viability of cells was tested by the neutral red assay.

### 2.8. Viability Tests

#### 2.8.1. Neutral Red Uptake Assay

The neutral red assay was performed to measure the cell viability. It was used to evaluate the cytotoxicity of the *E. camaldulensis* and CDDP on the A549 and MCF-7 carcinoma cells.

It is a spectrophotometric method that relies on the ability of the cell to take up and retain the dye. Lysosomes of non-viable cells are not able to do this and the dye diffuses out of the cell during incubation, and the spectrophotometric absorption values obtained are lower than for the viable cells.

The neutral red (NR) stock solution was prepared by measuring 40 mg of the NR powder and dissolving in 10 mL of ultra-pure water, such that the concentration is 4 mg/mL to obtain solution 1. Then, 100 μL of solution 1 were taken and added to a 100 mL medium (DMEM), thus obtaining solution 2. The medium was aspirated and cells were washed with 400 μL PBS. After that, 200 μL of solution 2 was added to each well followed by 3 h of incubation. Five milliliters of acetic acid + 95 mL of distilled water were prepared to obtain solution A. Eight milliliters of solution A + 7 mL of distilled water + 25 mL ethanol (70%) were mixed to obtain solution B. After 3 h, neutral red was taken out, and cells were washed again twice with PBS (400 μL), then 200 μL of solution B were added to each well. After 5 min, cells were transferred to a 96-well plate and the absorbance was measured at 450 nm using the ELISA technique.

#### 2.8.2. MTT Assay

The cells were seeded in 96-well plates with a density of 10 × 103 cells/well. The next day, the cells were treated with extracts at ascending concentrations (three wells for each concentration). After 24 h of treatment, the culture media containing the extracts were removed and the cells were incubated for 3 h in a 20-μL/well of a 0.2–0.5 mg/mL MTT solution. In living cells, the tetrazolium salt transformed into violet formazan by mitochondrial dehydrogenase, while dead cells lost the ability to convert MTT into formazan, thus color formation served as a useful and convenient marker of only the viable cells.

After incubation, a volume of 100-μL/well of a solubilization solution (11 g SDS, 50 mL isopropanol and 0.02 M HCL, 0.05 mg/mL) were added.

After 10 min in the dark, the optical density was measured in an ELISA plate reader at the wavelength of 570/690 nm. 

### 2.9. Statistical Analysis 

All presented results correspond to the mean ± standard deviation (SD). Statistical analysis was carried out by the mean of GraphPad Prism 5 (GraphPad Software Inc., San Diego, CA, USA).

A two-way analysis of variance (ANOVA) was employed to determine the p-values: * *p* < 0.05, ** *p* < 0.01, and *** *p* < 0.001. Duncan’s means comparison test was used to compare the different treatments performed at the *p* < 0.05 probability level.

## 3. Results

### 3.1. Chemical Testing Results

#### Extraction Yields of *E. camaldulensis* Crude Extracts

Table 2 shows the yield percentage of active compounds extracted from *E. camaldulensis*. The aqueous solvent has more ability to extract the active components from the plant than the ethanolic one.

Table 3 demonstrates the phytochemical screening of the two extracts (ethanol, aqueous) of *E. camaldulensis*. As shown in the table below, there is a qualitative difference between the ethanolic and aqueous extracts in terms of resins, cardiac glycosides, and saponins.

Important medicinal phytochemicals such as phenols, terpenoids, flavonoids, alkaloids, sterols and steroids, and fixed oil and fatty acids, were present in *E. camaldulensis*.

### 3.2. Chemical Quantifications of Secondary Products

#### Total Phenolic Content

The total phenolic content of both ethanolic and aqueous extracts of *E. camaldulensis* was estimated, where the aqueous extract contains more phenols than the ethanolic one (Table 4).

### 3.3. Antioxidant Activity (AA)

The transformation of the violet color to a yellow color indicates a high antioxidant activity, where the aqueous extract has a higher antioxidant activity compared to the ethanolic one (Table 3).

### 3.4. Cell Viability Tests on A549 and MCF-7 Cell Lines (Neutral Red Assay)

In order to determine the effect of *E. camaldulensis* extracts on A549 and MCF-7 cell lines, the neutral red assay was performed to measure the percentage of viability of cells:

#### 3.4.1. Treatment of A549 Cell Line with the Ethanolic Extract (EE)

**Figure 2:**
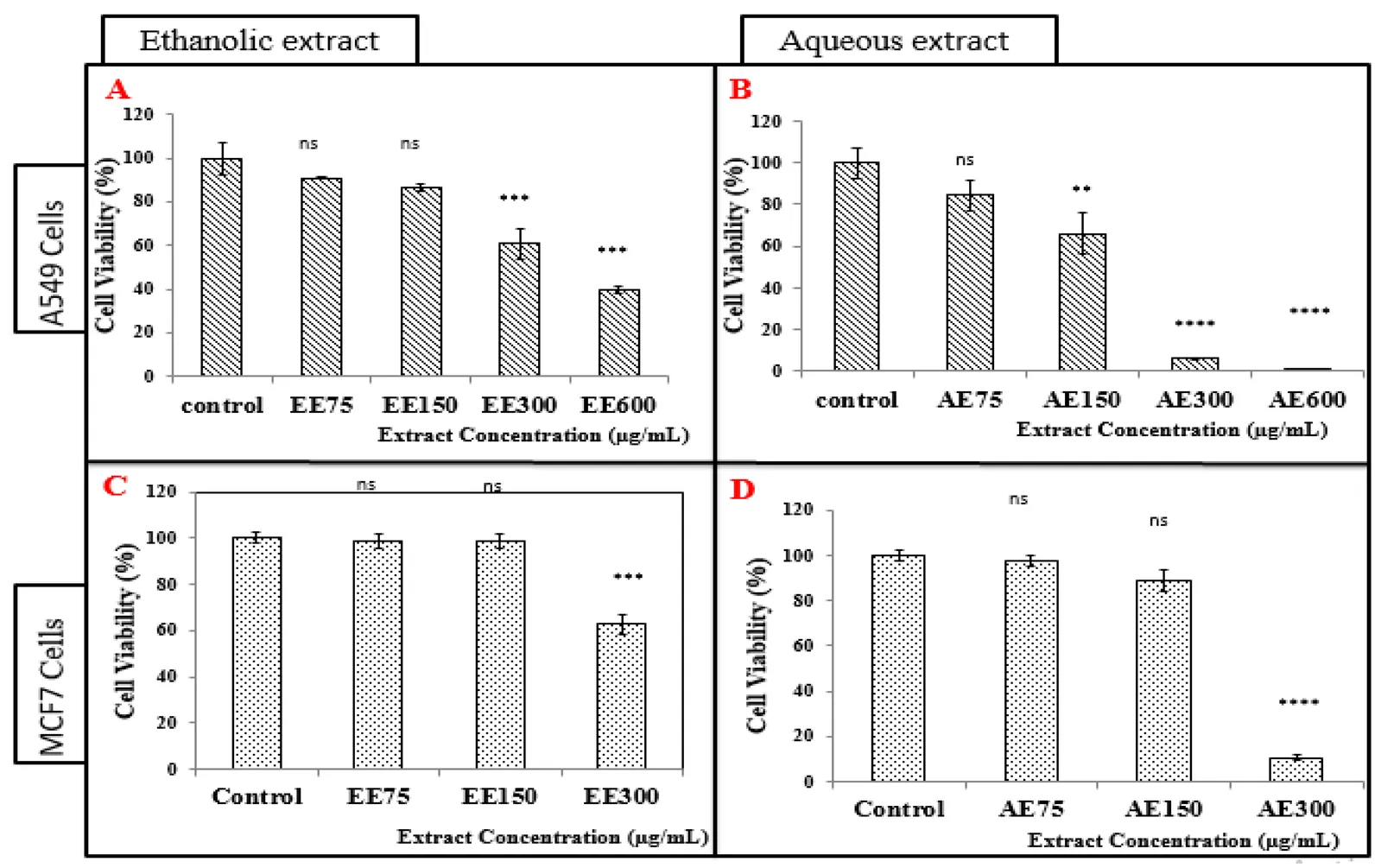
Cell viability of A549 and MCF-7 cell lines after treatment for 24 h with ethanolic extract (EE) and aqueous (AE). (**A**) Cell viability of A549 cells after treatment with increasing concentrations of ethanolic extract of *E. camaldulensis* for 24 h. (**B**) Cell viability of A549 cells after treatment with increasing concentrations of aqueous extract of *E. camaldulensis* for 24 h. (**C**) Cell viability of MCF-7 cells after treatment with increasing concentrations of ethanolic extract of *E. camaldulensis* for 24 h. (**D**) Cell viability of MCF-7 cells after treatment with increasing concentrations of aqueous extract of *E. camaldulensis* for 24 h (* *p* < 0.05, ** *p* < 0.01, *** *p* < 0.001, **** *p* < 0.001).

The treatment with the ethanolic extract of *E. camaldulensis* at different concentrations (75, 150, 300, and 600 µg/mL) showed a decrease in the cell viability of A549 cells by comparison to untreated cells (Figure 2A). No significant difference was observed at the concentration of 150 µg/mL in which cell viability was decreased by 10%. However, a significant decrease was observed for concentrations beyond 300 in comparison to the control which mirrored that the cell death caused by this extract was dose dependent. 

#### 3.4.2. Treatment of A549 Cell Line with the Aqueous Extract (AE)

The treatment with the aqueous extract of *E. camaldulensis* at different concentrations (75, 150, 300, and 600 µg/mL) induced a decrease in the viability of A549 cells (Figure 2B). A significant decrease was noticed with the concentration of 150 µg/mL (34%) which was three times lower than the result observed for the concentration at 600 µg/mL (98% death) by the comparison to the control. Therefore, treatment of A549 with the aqueous extract is dose dependent.

Expectedly, the aqueous extract was more toxic to A549 cells compared to the ethanolic one; where at the same concentration of both extracts, the % of cell viability was less after treatment with the aqueous extract compared to the ethanolic one; for example, at a concentration of 150 µg/mL, the percentage viability of A549 has decreased to 66% after the treatment with the aqueous extract, while in the case of ethanolic extract, this value reached 86.15%. 

This result allowed choosing the aqueous extract for the assays in combination with CDDP.

#### 3.4.3. Treatment of MCF-7 Cells with the Ethanolic Extract (EE)

The treatment with the ethanolic extract of *E. camaldulensis* at different concentrations (75, 150, and 300 µg/mL) showed a decrease in the % of cell viability of MCF-7 cells (Figure 2C). There is a significant decrease at the concentration of 300 µg/mL (38% death) by the comparison to the non-treated cells (control). Therefore, the death caused by this extract is dose dependent.

#### 3.4.4. Treatment of MCF-7 Cells with the Aqueous Extract (AE)

The treatment with the aqueous extract of *E. camaldulensis* at different concentrations (75, 150, and 300 µg/mL) showed a decrease in the % viability of MCF-7 cells. There is a significant decrease at the concentration of 300 µg/mL (89.5% death) by the comparison to the non-treated cells (control). Therefore, the treatment of MCF-7 cells with the aqueous extract is dose dependent (Figure 2D).

By comparing the results of the treatment of MCF-7 cells between the ethanolic and aqueous extracts, it is obvious that the aqueous extract was more toxic to MCF-7 cells compared to the ethanolic one; where at the same concentration of both extracts, for example 300 µg/mL, the % of cell viability was decreased six times by the aqueous extract more than the ethanolic one.

That is why the aqueous extract was chosen for the combination with CDDP.

Before combining the aqueous extract with CDDP, we determined the least toxic concentrations of CDDP to be used in the combination; the neutral red assay was performed to measure the percentage of viability of cells on A549 and MCF-7 cell lines.

### 3.5. Treatment of A549 and MCF-7 Cell Lines with CDDP 

#### 3.5.1. Treatment of A549 Cell Line with CDDP 

**Figure 3:**
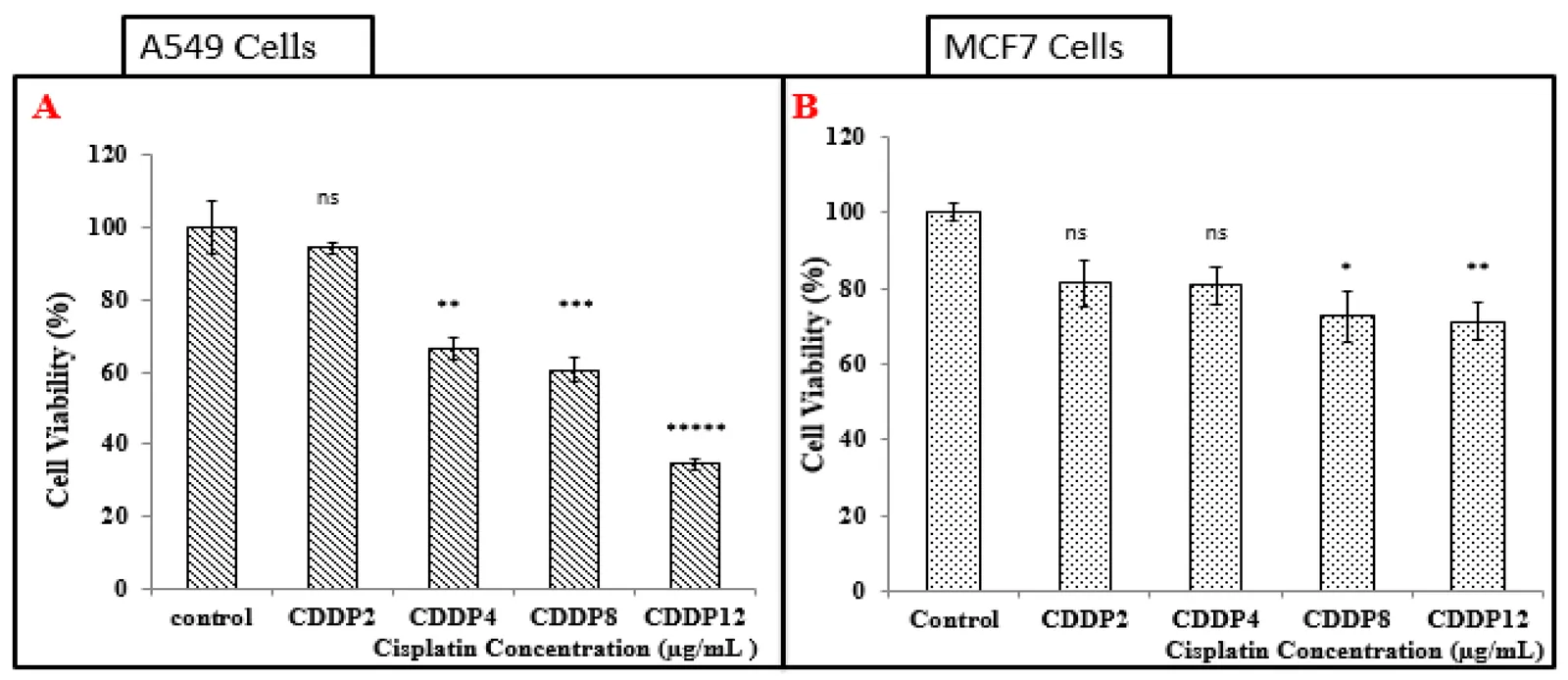
Cell viability of A549 and MCF-7 cell lines after treatment for 24 h with cis-dichloro-diamine-platinum (CDDP). (**A**) Cell viability of A549 cells after treatment with increasing concentrations of CDDP for 24 h. (**B**) Cell viability of MCF-7 cells after treatment with increasing concentrations of CDDP for 24 h (* *p* < 0.05, ** *p* < 0.01, *** *p* < 0.001, **** *p* < 0.001).

The mortality caused by CDDP was dose dependent (Figure 3A). Indeed, as the dose of CDDP increased, the cell viability decreased significantly compared to the control. Treatment with CDDP at the least concentration of 4 µg/mL for 24 h caused a significant decrease in cell viability compared to the control. 

#### 3.5.2. Treatment of MCF-7 Cells with CDDP

The mortality caused by CDDP is dose dependent. In other words, as the dose of CDDP increases the % of cell viability decreases significantly by comparison to the control (Figure 3B). A non-significant decrease appeared when the treated MCF-7 cells with CDDP at the concentration of 4 µg/mL was compared to the control.

However, this concentration (4 µg/mL) which is the least toxic one to A549 was chosen for later combination with the plant extract.

### 3.6. Treatment of A549 and MCF-7 Cell Lines with the Combination of Aqueous Extract (AE) and CDDP

#### 3.6.1. Treatment of A549 Cell Line with the Combination of Aqueous Extract (AE) and CDDP

According to the above results, we continued our experiments and we tested the cell viability of A549, MCF-7, and PBMC cells after treatment with the combination of the aqueous extract with low-dose CDDP.

**Figure 4:**
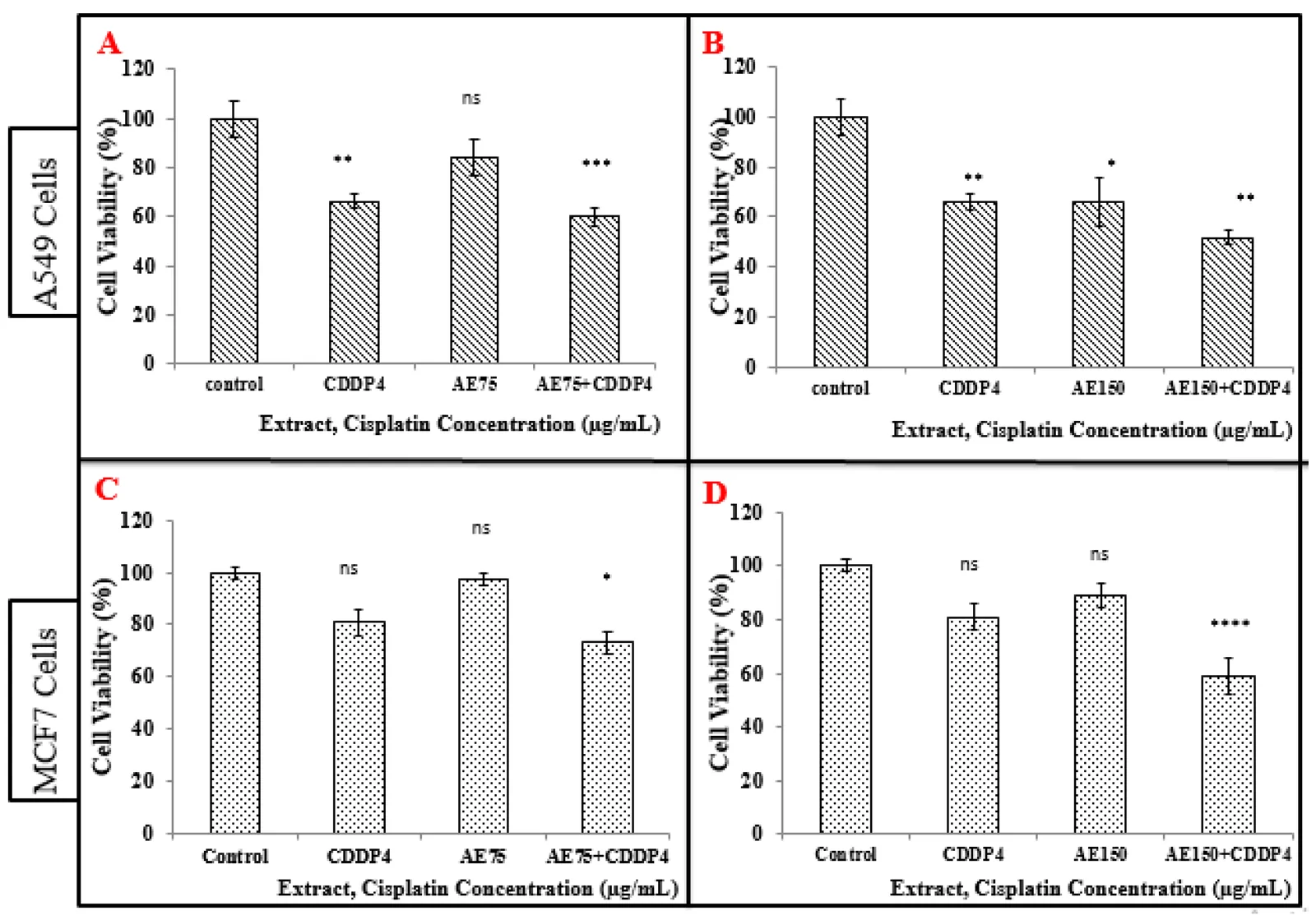
Cell viability of A549 and MCF-7 after treatment for 24 h. (**A**) Cell viability of A549 for 24 h after treatment with CDDP (4 µg/mL) combined with the aqueous extract (AE 75 µg/mL). (**B**) Cell viability of A549 for 24 h after treatment with CDDP (4 µg/mL) combined with the aqueous extract (AE 150 µg/mL). (**C**) Cell viability of MCF-7 for 24 h after treatment with CDDP (4 µg/mL) combined with the aqueous extract (AE 75 µg/mL). (**D**) Cell viability of MCF-7 for 24 h after treatment with CDDP (4 µg/mL) combined with the aqueous extract (AE 150 µg/mL) (* *p* < 0.05, ** *p* < 0.01, *** *p* < 0.001, **** *p* < 0.001).

There is a significant decrease observed in the viability of A549 cells after treatment, when combining CDDP (4 µg/mL) with the aqueous extract (75 µg/mL) in comparison to the control (Figure 4A). The cell viability observed in the combination of CDDP (4 µg/mL) with the aqueous extract (75 µg/mL) was less in comparison compared to either CDDP (4 µg/mL) or aqueous extract (75 µg/mL) separately.

In addition, a significant decrease in viability of A549 cells was observed after the treatment when combining CDDP (4 µg/mL) and the aqueous extract (150 µg/mL) in comparison to the control (Figure 4B). The past mentioned combination as a treatment was more efficient than using each one alone in treating the cells. Additionally, the decrease in cell viability was more significant when combining the aqueous solution of 150 µg/mL with CDDP 4 µg/mL (Figure 4B).

#### 3.6.2. Treatment of MCF-7 Cells with the Combination of Aqueous Extract (AE) and CDDP

A significant decrease in the cell viability of MCF-7 cells appeared after treatment either with a combination of CDDP (4 µg/mL) and the aqueous extract (75 µg/mL) (Figure 4C), and also with CDDP (4 µg/mL) and the aqueous extract (150 µg/mL) compared to the control. This combination was more efficient than using each treatment alone (Figure 4D). Moreover, this significant decrease was the greatest when combining specifically the aqueous 150 µg/mL with CDDP 4 µg/mL.

### 3.7. Cell Viability Tests on A549 Cell Line (MTT Assay)

**Figure 5:**
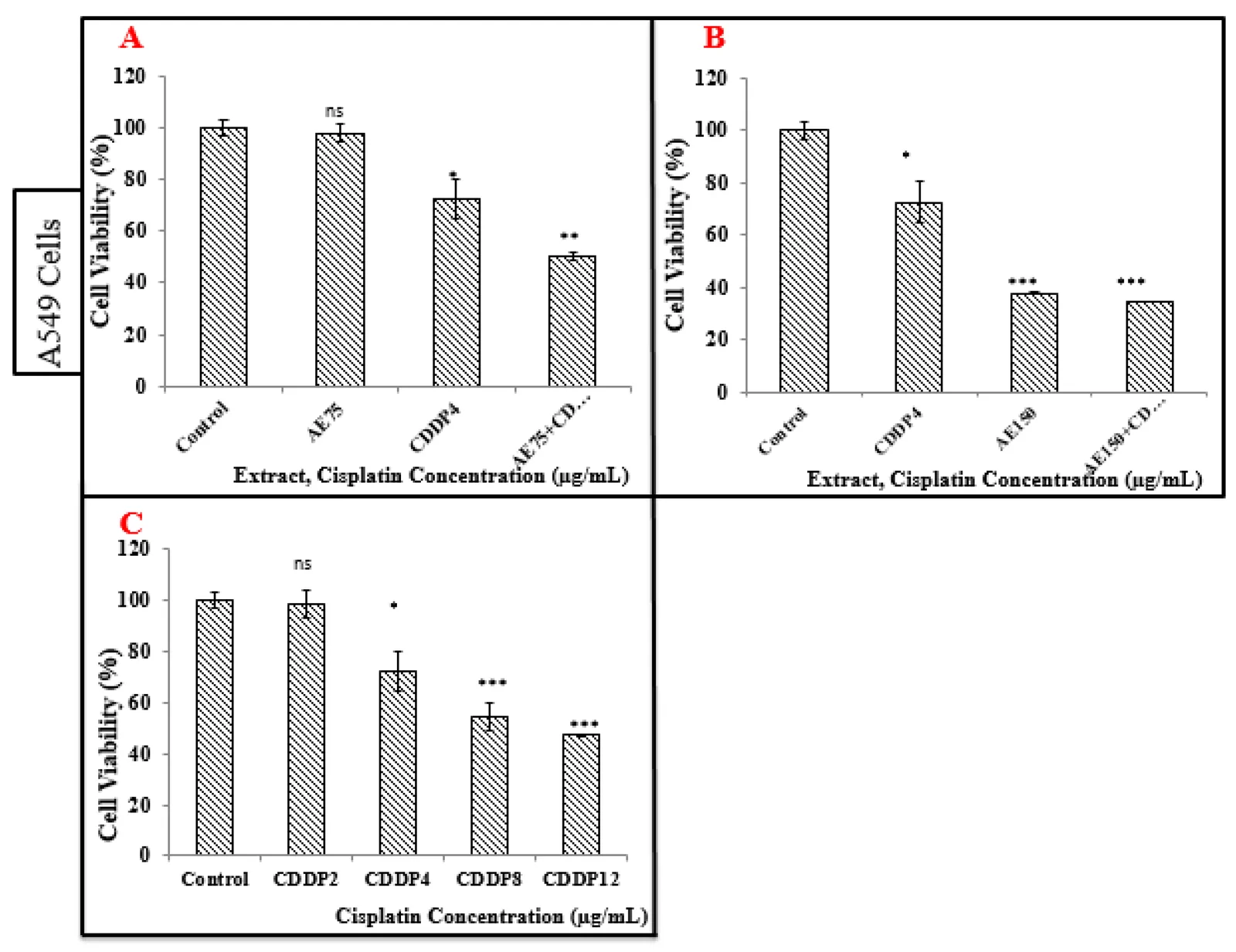
Cell viability of A549 after treatment for 24 h. (**A**) Cell viability of A549 for 24 h after treatment with CDDP (4 µg/mL) combined with the aqueous extract (AE 75 µg/mL), (MTT). (**B**) Cell viability of A549 for 24 h after treatment with CDDP (4 µg/mL) combined with the aqueous extract (AE 150 µg/mL), (MTT). (**C**) Cell viability of A549 cells after treatment with increasing concentrations of CDDP for 24 h (* *p* < 0.05, ** *p* < 0.01, *** *p* < 0.001, **** *p* < 0.001).

The results of the neutral red assay were confirmed by MTT assay as shown in Figure 5. A significant decrease was observed in the viability of A549 cells after treatment, when combining CDDP (4 µg/mL) with the aqueous extract (75 µg/mL) in comparison to the control (Figure 5A). The cell viability observed in the combination of CDDP (4 µg/mL) with the aqueous extract (75 µg/mL) was lower in comparison to either CDDP (4 µg/mL) or aqueous extract (75 µg/mL) separately. Moreover, after combining CDDP (4 µg/mL) and the aqueous extract (150 µg/mL), the viability of A549 cells decreased in comparison to the control (Figure 5B). 

### 3.8. Cell Viability Tests on Peripheral Blood Mononuclear Cells (PBMC) (Neutral Red Assay) 

The *E. camaldulensis* extract seems to have a potential therapeutic effect on A549 and MCF-7 cells. Therefore, it was selected to test its effect on the normal cells: Peripheral blood mononuclear cells (PBMC). 

#### 3.8.1. Treatment of PBMC with the Ethanolic Extract (EE)

**Figure 6:**
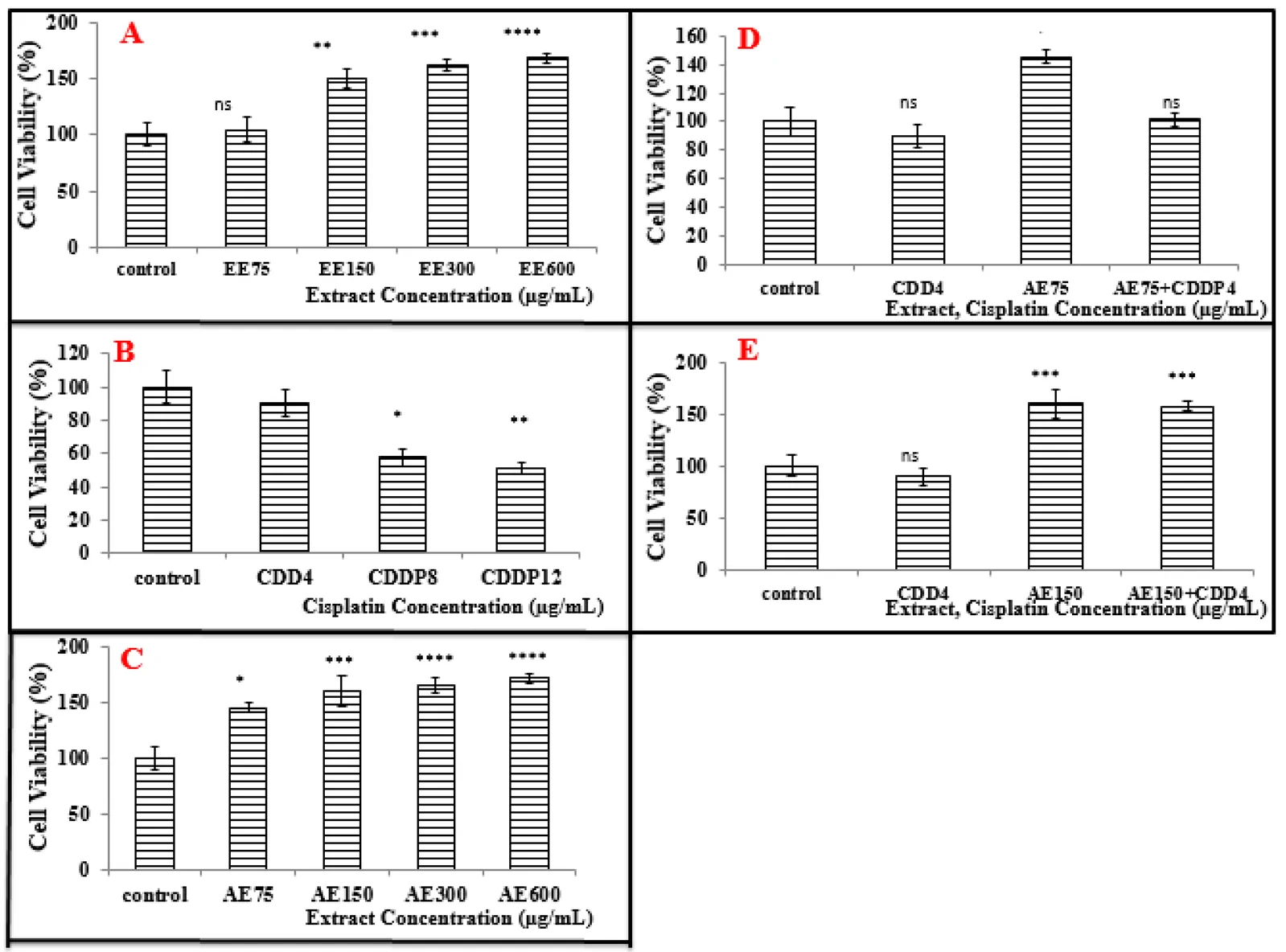
Cell viability of peripheral blood mononuclear cells (PBMC) after treatment for 24 h. (**A**) Cell viability of PBMC cells after treatment with the ethanolic extract of *E. camaldulensis* for 24 h. (**B**) Cell viability of PBMC cells after treatment with increasing concentrations of CDDP for 24 h. (**C**) Cell viability of PBMC cells after treatment with the aqueous extract of *E. camaldulensis* for 24 h. (**D**) Cell viability of PBMC 24 h after treatment with CDDP (4 µg/mL) combined with the aqueous extract (AE 75 µg/mL). (**E**) Cell viability of PBMC 24 h after treatment with CDDP (4 µg/mL) combined with the aqueous extract (AE 150 µg/mL) (* *p* < 0.05, ** *p* < 0.01, *** *p* < 0.001, **** *p* < 0.001).

The treatment with the ethanolic extract of *E. camaldulensis* at different concentrations (75, 150, 300, and 600 µg/mL) increased the % viability of PBMC by the comparison to the control. The ethanolic extract increased the % of cell viability of PBMC in a dose dependent manner. For example, at the concentration of 600 µg/mL the % viability was increased to 168% more than at 150 µg/mL which is 150% (Figure 6A).

#### 3.8.2. Treatment of PBMC with the Aqueous Extract (AE)

The treatment with the aqueous extract of *E. camaldulensis* at different concentrations (75, 150, 300, and 600 µg/mL) increased the % of cell viability of PBMC in a dose dependent manner. A significant increase in the % of cell viability from 145% to 171% appeared when increasing the concentration of the aqueous extract from 75 to 600 µg/mL, respectively (Figure 6C).

Moreover, it is obvious that the aqueous extract is more potent compared to the ethanolic one, that is why it was chosen for the combination with CDDP.

#### 3.8.3. Treatment of PBMC with CDDP

The mortality caused by CDDP is dose dependent. In other words, as the dose of CDDP increases the % of cell viability decreases significantly compared to the control. Treatment with CDDP at the concentration of 4 µg/mL for 24 h caused a non-significant decrease in the % of cell viability compared to the control, but this concentration which is the least toxic one to PBMC is chosen for a later combination with the plant extract (Figure 6B). 

#### 3.8.4. Treatment of PBMC with the Combination of Aqueous Extract (AE) and CDDP

No significant change in % of cell viability of PBMC was noticed after treatment with combinations of CDDP (4 µg/mL) and the aqueous extract (75 µg/mL) compared to the control (Figure 6D). However, there is a significant increase in the % of cell viability of PBMC after treatment with combinations of CDDP (4 µg/mL) and the aqueous extract (150 µg/mL) compared to the control as shown in Figure 6E.

## 4. Discussion

Globally, the increase in cancer incidence and mortality rates drives most researchers to do their best trying to find a treatment for this threat. Lung and breast cancers account for the highest cases and deaths, especially in undeveloped countries, such as Lebanon [23].

Many recent studies have been conducted on plants used in traditional medicine for treating various types of cancers, and antitumor compounds were found in them. 

As an illustration, in vitro studies proved that the ethanolic extract of *E. camaldulensis* leaf exerts a cytotoxic effect on human chronic myelogenous leukemia cells (k562cells) [24].

Another study confirmed that the essential oil extracted from leaves of *E. benthamii* showed a cytotoxic effect against Jurkat (J774A.1) (T leukemia cells) and HeLa cells lines (cervical cancer cells) [19].

Moreover, cytotoxic activities of volatile oils and extracts from stems, leaves, and flowers of *E. sideroxylon* and *E. torquata* against the human breast adenocarcinoma cell line (MCF-7) were shown [25].

In this study, qualitative and quantitative chemical analysis of active constituents of both extracts was performed. Then, the antioxidant activity of the ethanolic and aqueous extracts of *E. camaldulensis* was evaluated. Finally, the cytotoxic effect of the two different extracts alone or the aqueous extract combined with a low dose of chemotherapeutic drug (CDDP) on A549, MCF-7 cells, and PBMC cells was studied.

The phytochemical screening plays a good role in the estimation of possible involvement of this plant in the prevention or treatment of some diseases. In this project, qualitative analysis showed that there is a qualitative difference between the ethanolic and aqueous extracts in terms of resins, cardiac glycosides, and saponins. Important medicinal phytochemicals such as phenols, terpenoids, flavonoids, alkaloids, sterols and steroids, fixed oils, and fatty acids are present in *E. camaldulensis*. Terpenoids are anti-inflammatory, antiviral, inhibiting cholesterol synthesis, and antibacterial [26].

It was also shown that the aqueous extract has more content of phenols and flavonoids than the ethanolic extract, which permits determining the mechanism of action of the combination of aqueous extract with CDDP. Bacaba is an inborn palm natural product from Amazon local wealthy in polyphenolics. The test appeared that these phenolic extracts actuated apoptosis in MCF-7 cancer cells through the mitochondrial pathway. Capaces-6, -8, and -9 were activated when compared to the untreated control in a measurement’s subordinate way (p < 0.05). Inside these, caspace-9 appeared the most noteworthy activation. Since MCF-7 cells do not express caspace-3 and based on extra examinations on PARP-cleavage employing a caspace-9 inhibitor, the tests suggest that caspace-9 plays an imperative part within the apoptosis [27]. Moreover, it was proved that the aqueous extract has a higher DPPH radical scavenging activity than the ethanolic extract.

Concerning the neutral red assay, a significant decrease in the % of cell viability was observed at a low concentration of the aqueous extract (from 150 µg/mL) compared to the ethanolic one (from 300 µg/mL) during the treatment of A549 cells. For both extracts, the decrease in % of cell viability is dose dependent.

Moreover, the combination of aqueous extract with CDDP decreases the % of cell viability to a lower extent than the extract alone after 24 h of treatment at the level of A549 cells. The MTT results had confirmed those of neutral red, so that the combination of aqueous extract with CDDP decreases significantly the % viability of A549 cells.

For MCF-7 cells, a significant decrease in the % of cell viability was observed at a concentration of 300 µg/mL for both aqueous and ethanolic extracts. Moreover, combining the aqueous extract with CDDP decreases the % of cell viability to a lower extent than the extract alone after 24 h of treatment.

Given these points, it can be concluded that the aqueous extract has more cytotoxic effect than the ethanolic extract. Thus, it can be concluded that the secondary metabolite contents and antioxidant activities are closely related with the anti-tumor effect of plant extracts.

Regarding the cytotoxicity of *E. camaldulensis* extracts on normal cells (PBMC), it was shown that neither the ethanolic extract nor aqueous extract develop any toxicity towards PBMC. On the other hand, both extracts increase the % of cell viability of PBMC, with a greater extent for the aqueous extract. Moreover, the combination of CDDP with the aqueous extract showed a greater % of cell viability compared to CDDP alone. These results demonstrated that *E. camaldulensis* leaf extracts protect the peripheral blood mononuclear cells (monocytes and lymphocytes) from the cytotoxicity of CDDP and enhance their proliferation.

CDDP gets to be activated once it enters the cell. Within the cytoplasm the chloride particles on CDDP are uprooted by water molecules. This hydrolyzed item could be a strong electrophile that can respond with any nucleophile, counting the sulfhydryl groups on proteins and nitrogen benefactor molecules on nucleic acids. CDDP ties to the N7 responsive center on purine buildups and as such can cause deoxyribonucleic acid (DNA) damage in cancer cells, blocking cell division and coming about in apoptotic cell death [20].

Patients treated with CDDP, alone or in combination with other chemotherapeutic drugs, endure from the side effects. Consequently, the thought is to decrease the concentration of CDDP and compensate it by the combination with the extract, in order to minimize the side effects. The results showed that the combinations with *E. camaldulensis* extracts had a synergistic anticancer effect that increased the anticancer effect of low-dose CDDP alone. These results encouraged the investigators to fabricate pharmaceutical drugs containing *E. camaldulensis* leaf extracts as an active ingredient.

The characteristic phenols were able to cause the arrest of the cancer cell cycle, proposing that particular cell cycle administrative proteins are conceivably included in their intracellular component of activity. Specifically, the characteristic compounds were uncovered to be more dynamic in CDDP-resistant cells than in wild sorts, particularly inducing apoptotic death [28].

Many therapies are now available for the treatment of lung and breast cancer including chemotherapy, radiotherapy, and immunotherapy.

In order to minimize the side effects associated with these therapies, chemotherapy combinations with plant extracts have been used.

Medicinal plants are important for pharmacological research and drug development. They can complement the activity of the drugs used in traditional medicine and to which resistance develops in patients.

Indeed, our study has shown that the Eucalyptus contains active compounds such as polyphenols, alkaloids, tannins, flavonoids, sterols, steroids, fatty acids, and glycosides. Of which the alkaloid and phenols have had significant anti-cancer and antioxidants activities, then we can suggest that *E. camaldulensis* that contains phenol can induce apoptosis of cancer cells. In the same way, the flavonoids have effects as antimutagens and anti-tumors. They also have a protective effect against cancer by their effect on signal transduction in cell proliferation and angiogenesis [29].

The DPPH method was used to monitor the antioxidant activity of the extracts of plants. The results obtained showed that this plant had a high antioxidant activity.

Neutral red results demonstrated that *E. camaldulensis* extracts have an anti-proliferative effect on the viability of A549 and MCF-7 cells with a higher extent for the aqueous extract. Where the antitumor effect is represented by decreased viability of these cells in a dose dependent manner.

In addition, the combination of the *E. camaldulensis* with low-dose CDDP exhibits a synergistic inhibitory effect at certain levels against the cell viability of A549 and MCF-7 cells.

On the other hand, both plant extracts showed no cytotoxicity towards normal cells (PBMC), where both extracts increased the viability of these cells and enhanced their proliferation with a greater extent to the aqueous extract.

## 5. Conclusions

Our study showed that the Eucalyptus contains active compounds such as polyphenols, alkaloids, tannins, flavonoids, sterols, steroids, fatty acids, and glycosides which induced significant anticancer activities. 

Treatment with ethanolic and aqueous extracts from *E. camaldulensis* have an interesting anti-proliferative effect on the viability of A549 and MCF-7 cells with a higher extent for the aqueous extract where the antitumor effect is represented by decreased viability of these cells in a dose dependent manner by neutral red test.

In addition, the combination of the *E. camaldulensis* with low dose CDDP exhibits a synergistic inhibitory effect at certain levels against the cell viability of A549 and MCF-7 cells. 

On the other hand, both plant extracts showed no cytotoxicity towards normal cells (PBMC), where both extracts increased the viability of these cells and enhanced their proliferation with a greater extent to the aqueous extract. 

These results suggest studying the effect of combination therapy of *E. camaldulensis* with low-dose CDDP in vivo by injecting mice with A549 adenocarcinoma cells and breast cancer cells MCF-7.

## Figures and Tables

**Table 1:** Phytochemical used screening tests.

Plant Constituents	Extract	Reagent	Color
Reducing sugar	0.5 mL	1 mL water + 5–8 drops of Fehlings (A + B) + boil	Brick-red precipitate
Anthraquinones	1 mL	1 mL HCL (10%) + boil	Precipitate
Proteins and amino acids	1 mL	1 mL ninhydrin (0.25%) + boil	Blue color
Phlabotannins	1 mL	1 mL HCL (1%) + boil (5 min) + cooling	Red precipitate
Alkaloids	1 mL	Five drops of Dragendorff	Reddish orange precipitate/reddish brown or turbidity
Tannins	1 mL	Ferric chloride (FeCl_3_ 1%)	Blue color
Resins	1 mL	Acetone + small amount of water + agitation	Turbidity
Terpenoids	1 mL	2 mL chloroform + 3 mL concentrated sulphuric acid	Reddish brown color in the surface
Flavonoids	1 mL	5 mL potassium hydroxide (KOH 50%)	Yellow color
Quinones	1 mL	HCL concentrated	Precipitate or yellow color
Sterols and Steroids	1 mL	2 mL chloroform + concentrated sulphuric acid	Red color of the upper layer + greenish yellow fluorescence in the acid layer
Diterpenes	1 mL dissolved in water	Few drops of copper sulphate	Green color
Anthocyanins	1 mL	1 mL NaOH (10%)	Blue color
Flavanones	1 mL	1 mL concentrated sulphuric acid	Purple red color
Lignines	2 mL	Safranine	Pink color
Cardiac glycosides	2 mL	1 mL acetic acid glacial + 1 drop ferric chloride FeCl_3_ (5%) + 1 mL concentrated sulphuric acid	Purple ring + brown ring + green ring
Saponins	2 mL	Vigorous shaking (5 min on Vortex)	Layer of foam
Phenols	5 mL	1 mL FeCl_3_ (1%) + 1 mL K_3_(Fe(CN)_6_) (1%)	Greenish blue color
Fixed oils and Fatty acids	Small amount of extract	On filter paper	Oil spot

**Table 2:** Extraction yields of the *E. camaldulensis* crude extract with two different solvents.

Solvent	Initial Weight (g)	Final Weight (g)	Extraction Yield (%)
Ethanol	20.0	3.3	16.5
Water	20.0	4.0	20.0

**Table 3:** Phytochemical screening tests of the ethanolic and aqueous extracts of *E. camaldulensis.*

Plant Constituents	Ethanol	Aqueous
Reducing sugar	+	+
Anthraquinones	+	+
Proteins and amino acids	-	-
Phlabotannins	-	-
Alkaloids	+	+
Tannins	+	+
Resins	+	-
Terpenoids	+	+
Flavonoids	+	+
Quinones	+	+
Sterols and Steroids	+	+
Diterpenes	+	+
Anthocyanins	-	-
Flavanones	+	+
Lignines	+	+
Cardiac glycosides	+	-
Saponins	-	+
Phenols	+	+
Fixed oils	+	+

**Table 4:** Chemical traits of two different extracts of *E. camaldulensis* products.

Solvent	TPC (mg GAE/g of Extract)	Soluble Sugar Test (%)	Dry matter (%)	Antioxidant Activity (TE Umol/g of Extract)
Ethanol	15.33 ± 0.54	9.95	95.06	211.89 ± 0.39
Aqueous	19.52 ± 0.22	9.95	95.06	214.01 ± 1.66
