# Correction: Nasser et al. Potency of Combining *Eucalyptus camaldulensis* subsp. *camaldulensis* with Low-Dose Cisplatin in A549 Human Lung Adenocarcinomas and MCF-7 Breast Adenocarcinoma. *Medicines* 2020, *7*, 40

**DOI:** 10.3390/medicines13030023

**Published:** 2026-07-14

**Authors:** Mohamad Nasser, Raghida Damaj, Othmane Merah, Akram Hijazi, Christine Trabolsi, Nour Wehbe, Malak Nasser, Batoul Al-Khatib, Ziad Damaj

**Affiliations:** 1 Plateforme de Recherche et d’Analyse en Sciences de l’Environnement (EDST-PRASE), Beirut P.O. Box 5, Lebanon; mohamed.nasser@ul.edu.lb (M.N.); rdamaj82@hotmail.com (R.D.); nourwehbi6@gmail.com (N.W.); malak-nasser1994@hotmail.com (M.N.); batool.khatib93@hotmail.com (B.A.-K.); damajz@hotmail.fr (Z.D.); 2 Laboratoire de Chimie Agroindustrielle, Université de Toulouse, INRA, 31030 Toulouse, France; 3 Département Génie Biologique, IUT A, Université Paul Sabatier, 24 rue d’Embaquès, 32000 Auch, France; 4 Faculty of Medicine, Lebanese University, Beirut P.O. Box 5, Lebanon; christinetrabolsi9@gmail.com

## Text Correction

There was an error in the original publication [1]. Non-standard terms were used, including “sulfhydryl bunches” and “deoxyribonucleic corrosive (DNA) harm”.

A correction has been made to Section 4. “Discussion”. The corrected sentence is below:

This hydrolyzed item could be a strong electrophile that can respond with any nucleophile, counting the sulfhydryl groups on proteins and nitrogen benefactor molecules on nucleic acids. CDDP ties to the N7 responsive center on purine buildups and as such can cause deoxyribonucleic acid (DNA) damage in cancer cells, blocking cell division and coming about in apoptotic cell death.

## References Correction

The references of the published paper [1] have been corrected. References numbered 1, 2, 3, 7, 15, and 17 have been replaced with the following references:

1. Siegel, R.L.; Miller, K.D.; Jemal, A. Cancer Statistics, 2018. *CA Cancer J. Clin.* 2018, *68*, 7–30. https://doi.org/10.3322/caac.21442.

2. Siegel, R.L.; Miller, K.D.; Jemal, A. Cancer Statistics, 2019. *CA Cancer J. Clin*. 2019, *69*, 7–34. https://doi.org/10.3322/caac.21551.

3. Ferlay, J.; Shin, H.R.; Bray, F.; Forman, D.; Mathers, C.; Parkin, D.M. Estimates of Worldwide Burden of Cancer in 2008: GLOBOCAN 2008. *Int. J. Cancer* 2010, *127*, 2893–2917. https://doi.org/10.1002/ijc.25516.

7. Aupérin, A.; Le Péchoux, C.; Rolland, E.; Curran, W.J.; Furuse, K.; Fournel, P.; Belderbos, J.; Clamon, G.; Ulutin, H.C.; Paulus, R.; et al. Meta-Analysis of Concomitant Versus Sequential Radiochemotherapy in Locally Advanced Non-Small-Cell Lung Cancer. *J. Clin. Oncol.* 2010, *28*, 2181–2190. https://doi.org/10.1200/JCO.2009.26.2543.

15. Coppen, J.J.W. (Ed.) *Eucalyptus: The Genus Eucalyptus*; CRC Press: London, UK, 2002. https://doi.org/10.1201/9780203219430.

17. Dhakad, A.K.; Pandey, V.V.; Beg, S.; Rawat, J.M.; Singh, A. Biological, Medicinal and Toxicological Significance of Eucalyptus Leaf Essential Oil: A Review. *J. Sci. Food Agric.* 2018, *98*, 833–848. https://doi.org/10.1002/jsfa.8600.

The authors state that the scientific conclusions are unaffected. This correction was approved by the Academic Editor. The original publication has also been updated.
